# Prognostic Nutritional Index, Controlling Nutritional Status (CONUT) Score, and Inflammatory Biomarkers as Predictors of Deep Vein Thrombosis, Acute Pulmonary Embolism, and Mortality in COVID-19 Patients

**DOI:** 10.3390/diagnostics12112757

**Published:** 2022-11-11

**Authors:** Adrian Vasile Mureșan, Ioana Hălmaciu, Emil Marian Arbănași, Réka Kaller, Eliza Mihaela Arbănași, Ovidiu Aurelian Budișcă, Răzvan Marian Melinte, Vlad Vunvulea, Rareș Cristian Filep, Lucian Mărginean, Bogdan Andrei Suciu, Klara Brinzaniuc, Raluca Niculescu, Eliza Russu

**Affiliations:** 1 Clinic of Vascular Surgery, Mures County Emergency Hospital, 540136 Targu Mures, Romania; 2 Department of Vascular Surgery, George Emil Palade University of Medicine, Pharmacy, Science, and Technology of Targu Mures, 540139 Targu Mures, Romania; 3 Department of Anatomy, George Emil Palade University of Medicine, Pharmacy, Science, and Technology of Targu Mures, 540139 Targu Mures, Romania; 4 Department of Radiology, Mureș County Emergency Hospital, 540136 Targu Mureș, Romania; 5 Doctoral School of Medicine and Pharmacy, George Emil Palade University of Medicine, Pharmacy, Science, and Technology of Targu Mures, 540142 Targu Mures, Romania; 6 Faculty of Pharmacy, George Emil Palade University of Medicine, Pharmacy, Science, and Technology of Targu Mures, 540139 Targu Mures, Romania; 7 Department of Surgery, George Emil Palade University of Medicine, Pharmacy, Science, and Technology of Targu Mures, 540139 Targu Mures, Romania; 8 Department of Orthopedics, Regina Maria Health Network, 540098 Targu Mures, Romania; 9 Department of Orthopedics, Humanitas MedLife Hospital, 400664 Cluj Napoca, Romania; 10 Department of Pathophysiology, George Emil Palade University of Medicine, Pharmacy, Science, and Technology of Targu Mures, 540139 Targu Mures, Romania

**Keywords:** monocyte to lymphocyte ratio, neutrophil to lymphocyte ratio, platelet to lymphocyte ratio, systemic inflammatory index, systemic inflammation response index, aggregate index of systemic inflammation, prognostic nutritional index, CONUT Score, COVID-19, pulmonary embolism

## Abstract

Background: Numerous tools, including nutritional and inflammatory markers, have been evaluated as the predictors of poor outcomes in COVID-19 patients. This study aims to verify the predictive role of the prognostic nutritional index (PNI), CONUT Score, and inflammatory markers (monocyte to lymphocyte ratio (MLR), neutrophil to lymphocyte ratio (NLR), platelet to lymphocyte ratio (PLR), systemic inflammatory index (SII), Systemic Inflammation Response Index (SIRI), and Aggregate Index of Systemic Inflammation (AISI)) in cases of deep vein thrombosis (DVT) and acute pulmonary embolism (APE) risk, as well as mortality, in COVID-19 patients. Methods: The present study was designed as an observational, analytical, retrospective cohort study, and included 899 patients over the age of 18 who had a COVID-19 infection, confirmed through real time-polymerase chain reaction (RT-PCR), and were admitted to the County Emergency Clinical Hospital and Modular Intensive Care Unit of UMFST “George Emil Palade” of Targu Mures, Romania between January 2020 and March 20212. Results: Non-Surviving patients were associated with a higher incidence of chronic kidney disease (*p* = 0.01), cardiovascular disease (atrial fibrillation (AF) *p* = 0.01; myocardial infarction (MI) *p* = 0.02; peripheral arterial disease (PAD) *p* = 0.0003), malignancy (*p* = 0.0001), tobacco (*p* = 0.0001), obesity (*p* = 0.01), dyslipidemia (*p* = 0.004), and malnutrition (*p* < 0.0001). Multivariate analysis showed that both nutritional and inflammatory markers had a high baseline value and were all independent predictors of adverse outcomes for all enrolled patients (for all *p* < 0.0001). The presence of PAD, malignancy, and tobacco, were also independent predictors of all outcomes. Conclusions: According to our findings, higher MLR, NLR, PLR, SII, SIRI, AISI, CONUT Score, and lower PNI values at admission strongly predict DVT risk, APE risk, and mortality in COVID-19 patients. Moreover, PAD, malignancy, and tobacco, all predicted all outcomes, while CKD predicts APE risk and mortality, but not the DVT risk.

## 1. Introduction

From December 2019 until now (16 October 2022), the Coronavirus disease (COVID-19) has affected over 629 million people and caused 6,571,064 deaths worldwide, having a negative impact on the medical practice [1,2,3].

COVID-19 patients’ clinical picture ranges from common symptoms such as fever, cough, and loss of taste and smells, to severe forms with a negative evolution, such as acute respiratory failure or sepsis. These also necessitate hospitalization in an Intensive Care Unit (ICU) and invasive mechanical ventilation (IMV) [4,5,6,7,8,9,10,11].

Among the most severe complications of COVID-19 patients are the hypercoagulability status, associated with arterial and venous thrombosis, which significantly increases the risk of mortality [12,13,14,15,16].

Thromboembolic complications occur at different rates depending on the severity of the disease, ranging from 10% for patients admitted to non-intensive care units, to 20%–30% for patients admitted to the ICU, and up to 70% for severe forms of the disease that require prolonged IMV [12,13,14,15,16]. Deep vein thrombosis (DVT) and acute pulmonary embolism (APE) are the most common thromboembolic consequences, both of which are associated with poor patient outcomes and a high mortality rate [12,17,18,19,20,21,22,23,24].

Numerous papers aimed to discover diagnostic and prognostic methods that could predict the deterioration of COVID-19 patients’ health. The prognostic role of D-Dimer, C-reactive protein (CRP), and fibrinogen levels in the incidence of thromboembolic events and mortality in COVID-19 patients is well-known and has been extensively researched in the specialized literature over the last two years [25,26,27,28,29,30,31].

Researchers have also focused on red blood cell ratios, which are based on the total amount of neutrophils, monocytes, platelets, and lymphocytes, which are simple to compute, and frequently conducted in hospitals.

Furthermore, the predictive role of these markers has been proven in the cases of renal pathologies [32,33,34], cardiovascular pathologies [35,36,37,38,39,40], oncological pathologies [41,42,43,44], and, most recently, for COVID-19 patients [45,46,47,48,49,50,51].

In terms of nutritional status, the prognostic nutritional index (PNI) and controlling nutritional status (CONUT) scores are associated with disease severity and have a predictive role in COVID-19 patient mortality [52,53,54,55,56,57]

The aims of this study were as follows: (1) to determine the role of systemic inflammatory biomarkers in DVT risk, APE risk, and mortality; (2) to determine the role of nutritional markers in DVT risk, APE risk, and mortality; and (3) to evaluate the risk factors associated with DVT, APE, and mortality in COVID-19 patients.

## 2. Materials and Methods

### 2.1. Study Design

The present study was designed as an observational, analytical, retrospective cohort study, and included 889 patients over 18 years of age with a diagnosis of COVID-19 infection, confirmed through real-time-polymerase chain reaction (RT-PCR) admitted to Mures County Emergency Hospital, in Targu Mures, Romania, and Modular Intensive Care Unit of George Emil Palade University of Medicine, Pharmacy, Science, and Technology of Targu Mures, Romania, between January 2020 and March 2022.

Exclusion criteria were as follows: patients with hematological diseases, autoimmune diseases, patients who needed ICU admission in the first 24 h, patients who developed other thromboembolic events during hospitalization, such as acute limb ischemia or stroke, and patients with a history of DVT or APE in the last year.

Data analysis was conducted depending on survival during the hospitalization and patients were divided into two groups named “Survivors” and “non-Survivors”. The ideal cut-off value for all markers studied was used to calculate the DVT and APE developed, and the mortality rate.

### 2.2. Data Collection

The patient’s age, gender, and hospitalization period were extracted from the hospital’s electronic database. Regarding comorbidities, the following cardiac pathologies were recorded: arterial hypertension (AH), atrial fibrillation (AF), ischemic heart disease (IHD), history of myocardial infarction (MI), chronic heart failure (CHF), as well as other pathologies: chronic kidney disease (CKD), peripheral arterial disease (PAD), and diabetes mellitus (DM).

The following were extracted from the first laboratory analyses: hemoglobin level, hematocrit level, glucose level, total cholesterol level, triglyceride level, serum albumin, Glomerular filtration rate (GFR), blood urea nitrogen (BUN), creatinine, number of neutrophils, lymphocytes, monocytes, platelets, potassium, and sodium.

All patients received prophylactic anticoagulation during the hospitalization with low-weight molecular heparin.

### 2.3. Nutritional and Inflammatory Markers

Nutritional and inflammatory biomarkers were determined from the first blood test result. The ratio was calculated using the equations as seen in Table 1.

### 2.4. Study Outcomes

The primary endpoints were the occurrence of DVT, APE, and mortality during the hospitalization stay. The number of days spent in the hospital and the combinate endpoint of DVT and APE were recorded as secondary outcomes. The primary outcomes were stratified for the optimal cut-off value of inflammatory and nutritional biomarkers.

### 2.5. Deep Vein Thrombosis and Acute Pulmonary Embolism Diagnostics

The patients who presented symptoms of DVT were evaluated by Doppler ultrasound for both upper or lower extremities, and the level of vein thrombosis was recorded. Moreover, patients with suspected APE during hospitalization were evaluated by using a Computed Tomography Angiogram.

### 2.6. Statistical Analysis

SPSS for Mac OS version 28.0.1.0 was used for statistical analysis (SPSS, Inc., Chicago, IL, USA). Chi-square tests were used to assess the associations of the ratios with category factors, while t-Student or Mann–Whitney tests were used to assess differences in continuous variables. To analyze the predictive power and to establish the cut-off values of inflammatory biomarkers, the receiver operating characteristic (ROC) curve analysis was utilized. The ROC curve analysis was used to determine the appropriate MLR, NLR, PLR, SII, SIRI, AISI, PNI, and CONUT Score cut-off values based on the Youden index (Youden Index = Sensitivity + Specificity – 1, ranging from 0 to 1). To identify independent predictors of DVT and APE risk, and mortality in COVID-19 patients, a multivariate logistic regression analysis using variables with *p* < 0.1 was undertaken.

## 3. Results

During the studied period, 889 patients were enrolled. Regarding the negative evolution, 143 patients (16.08%) died during hospitalization, 191 patients (21.48%) had DVT, 62 patients had APE (6.97%), 38 patients (4.27%) were diagnosed with DVT and APE. Of the patients, 474 (53.32%) were male, with a mean age of 70.5 ± 12.9 (21 to 101).

Regarding the comorbidities and risk factors, the non-survivor patients had a higher incidence of AF (*p* = 0.01), MI (*p* = 0.02), CKD (*p* = 0.01), PAD (*p* = 0.001), Tobacco (*p* = 0.0001), Obesity (*p* = 0.01), and dyslipidemia (*p* = 0.004), as seen in Table 2. In terms of Nutritional status, lower PNI score (*p* < 0.0001), higher CONUT Score (*p* < 0.0001), as well as moderate (*p* < 0.0001) and severe (*p* < 0.0001) malnutrition were present in the non-survivors group. Moreover, regarding the laboratory findings, non-survivor patients had lower hemoglobin levels (*p* = 0.001), hematocrit (*p* = 0.0002), cholesterol (*p* < 0.0001), albumin (*p* < 0.0001), and lymphocyte (*p* < 0.0001), and higher glucose (*p* = 0.006), bun (*p* < 0.0001), creatinine (*p* < 0.0001), and neutrophils (*p* < 0.0001). All systemic inflammatory markers were higher in the second group (for all *p* < 0.0001). Moreover, all outcomes studied were higher in the poor outcome group (for all *p* < 0.0001).

**Figure 1:**
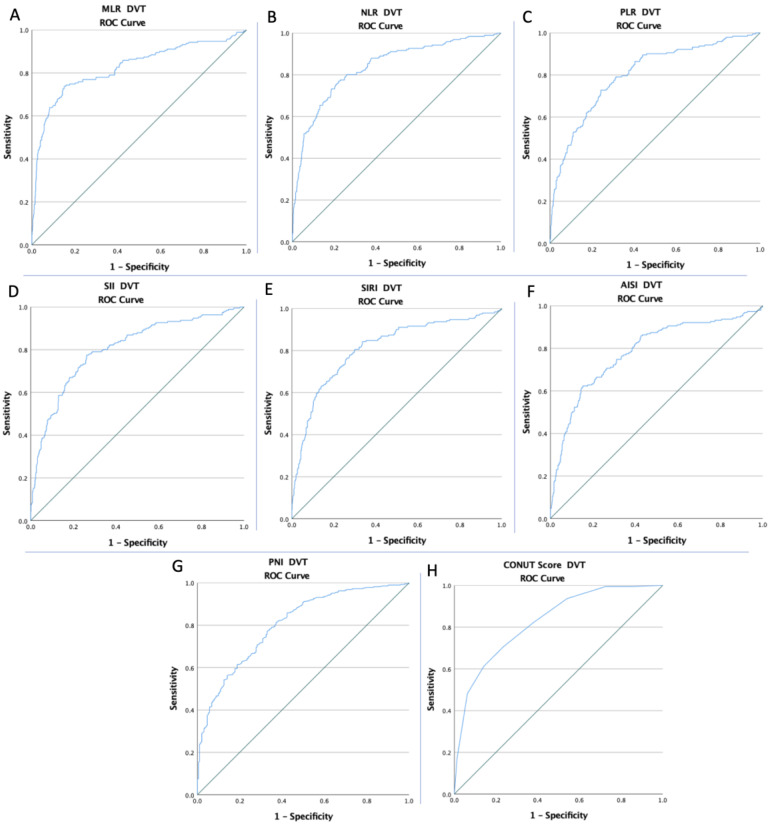
The ROC curve analysis concerning DVT risk (**A**) for the MLR (AUC: 0.824; *p* < 0.0001), (**B**) for the NLR (AUC: 0.836; *p* < 0.0001), (**C**) for the PLR (AUC: 0.802; *p* < 0.0001), (**D**) for the SII (AUC: 0.805; *p* < 0.0001), (**E**) for the SIRI (AUC: 0.811; *p* < 0.0001), (**F**) for the AISI (AUC: 0.784; *p* < 0.0001), (**G**) for the PNI (AUC: 0.803; *p* < 0.0001), and (**H**) for the CONUT Score (AUC: 0.826; *p* < 0.0001).

**Figure 2:**
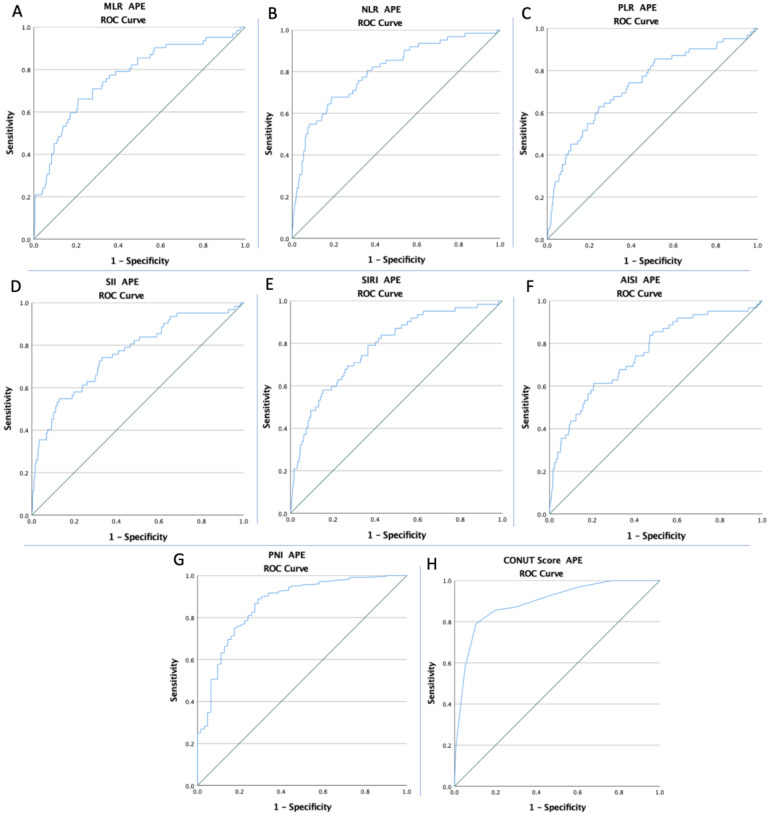
The ROC curve analysis concerning APE risk (**A**) for the MLR (AUC: 0.766; *p* < 0.0001), (**B**) for the NLR (AUC: 0.801; *p* < 0.0001), (**C**) for the PLR (AUC: 0.734; *p* < 0.0001), (**D**) for the SII (AUC: 0.761; *p* < 0.0001), (**E**) for the SIRI (AUC: 0.783; *p* < 0.0001), (**F**) for the AISI (AUC: 0.750; *p* < 0.0001), (**G**) for the PNI (AUC: 0.864; *p* < 0.0001), and (**H**) for the CONUT Score (AUC: 0.892; *p* < 0.0001).

**Figure 3:**
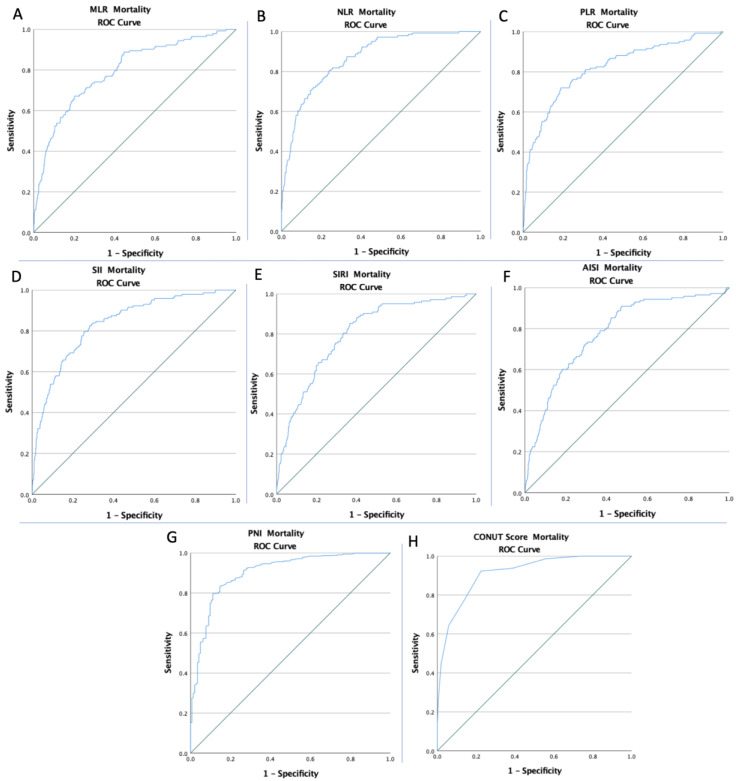
The ROC curve analysis concerning mortality (**A**) for the MLR (AUC: 0.794; *p* < 0.0001), (**B**) for the NLR (AUC: 0.868; *p* < 0.0001), (**C**) for the PLR (AUC: 0.819; *p* < 0.0001), (**D**) for the SII (AUC: 0.836; *p* < 0.0001), (**E**) for the SIRI (AUC: 0.801; *p* < 0.0001), (**F**) for the AISI (AUC: 0.780; *p* < 0.0001), (**G**) for the PNI (AUC: 0.903; *p* < 0.0001), and (**H**) for the CONUT Score (AUC: 0.913; *p* < 0.0001).

The ROC curves of all inflammatory and nutritional markers were created to determine whether the baseline of these markers was predictive of DVT risk, APE risk, and mortality during the hospitalization stay (Figure 1, Figure 2 and Figure 3). The optimal cut-off value obtained from Youden’s index, areas under the curve (AUC), and the predictive accuracy of the markers are listed in Table 3.

The DVT, APE risk, and mortality were further analyzed after dividing the patients into paired groups, according to the optimal cut-off value of all studied markers. Moreover, there was a higher incidence of all outcomes for all the markers as seen in Table 4.

A multivariate analysis was used to determine the association between all markers, underlying risk factors, DVT, APE development risk, and mortality during the hospitalization. A high baseline value of all systemic inflammatory markers and CONUT Score was a strong independent predictor of all outcomes (for all *p* < 0.0001), as well as a lower baseline value of PNI (*p* < 0.0001). Moreover, as shown in Table 5, PAD (OR:2.10 *p* < 0.001; OR:2.12 *p* = 0.005; and OR:1.92 *p* < 0.001), malignancy (OR:2.70 *p* <0.001; OR:4.55 *p* < 0.002; and OR:2.42 *p* = 0.001), and tobacco (OR:2.01 *p* < 0.001; OR:2.32 *p* = 0.02; and OR:2.10 *p* < 0.001) were predictors of all outcomes. Furthermore, CKD (OR:1.95 *p* = 0.02 and OR:1.77 *p* = 0.01) was a predictor of APE risk and mortality (Table 5).

## 4. Discussion

The main result of this study is that inflammatory and nutritional indicators might predict the risk of DVT and APE, as well as death during hospitalization in COVID-19 patients. Moreover, BAP, malignancy, and tobacco were strong predictors of the three outcomes, and CKD was a predictor of APE risk and mortality. To our knowledge, this is the first study that analyzes all hematological markers (MLR, NLR, PLR, SII, SIRI, and AISI), and nutritional markers (PNI and CONUT Score), in the prediction of DVT, APE, and mortality, on 889 COVID-19 patients.

Numerous research studies have been published in the last two years on the predictive significance of inflammatory markers and the mortality of COVID-19 patients [45,46,48,49,50,51,58,59,60,61,62]. Among the analyzed markers, NLR has the greatest interest for researchers, whose optimal cut-off value varies between 6.883 and 11.57 [48,50,51,58,59,60]. Thus, in the work published by Citu et al. [48], which included 108 patients, it was demonstrated that NLR > 9.1 is a predictor of mortality (HR:3.85; *p* = 0.01), and the work published by Kudlinski et al. [51], resulted in the association of NLR > 11.57 (HR:2.12; *p* = 0.008) with mortality in the case of 285 COVID-19 patients. Additionally, Rose et al. [58] concluded that NLR > 11.38 is associated with mortality (OR: 1.82; *p* = 0.01), in a study conducted on a group of 454 COVID-19 patients. In terms of MLR, studies by Halmaciu et al. [45], Arbanasi et al. [46], and Citu et al. [48], resulted in the association of MLR > 0.54 (OR:6.49; *p* < 0.001), MLR > 0.45 (OR:5.51; *p* < 0.001) and MLR > 0.69 (HR:3.05; *p* = 0.02), respectively, with mortality.

In regard to the predictive role of nutritional markers, in the meta-analysis published by Hung et al. [52], in which 13 studies were included, and a total number of 4204 COVID-19 patients, it was demonstrated that PNI is a predictor of mortality. Moreover, research by Bodolea et al. [53] resulted in PNI > 28.05 (HR:0.91; *p* = 0.01), and a CONUT Score > 7.5 (HR:1.15; *p* = 0.01) becoming associated with mortality, in the case of 90 COVID-19 patients with the severe form.

In terms of all inflammatory and nutritional markers’ optimal cut-off value regarding the non-COVID APE patients, Efros et al. [38] demonstrated that an NLR > 5.12 (OR:2.82; *p* < 0.001) and malignancy (OR:1.72; *p* < 0.001) are predictors of 30-day mortality in 2072 patients with APE. Moreover, Yildirim et al. [63] concluded that a CONUT Score > 4 (OR:1.39; *p* = 0.01) was an independent predictor of in-hospital mortality, in 308 consecutive non-COVID patients. Also, Hayiroglu et al. [64] demonstrated that PNI > 38 (AUC:0.79; *p* < 0.001) is an independent prognostic factor for survival in 251 patients with APE.

Numerous recent studies have also shown the superior role of superficial and deep learning-based intelligence in the diagnosis of COVID-19 patients using X-rays and CT scans with higher accuracy than the standard diagnostic approach [65,66,67,68,69,70].

This work supplements the previous two studies published by our group of researchers, Arbanasi et al. and Halmaciu et al., which revealed the role of inflammatory markers in the prediction of ICU admission, IMV necessity, ALI risk, and mortality [45,46]. The hypercoagulability status of COVID-19 patients is well known, and the unpredictable evolution of these fragile patients, as well as the presence of comorbidities and risk factors, requires the establishment of prognostic tools and stratification of patients for better management of their health.

As established in our two previous studies, systemic inflammatory biomarkers based on the total number of neutrophils, monocytes, lymphocytes, and platelets outperform traditional inflammatory indicators including D-Dimers, interleukin-6, and fibrinogen. They are also commonly tested and have a low cost when compared to other inflammatory markers.

Among the study’s strengths are the inclusion of all hematological markers based on red cell blood, as well as nutritional markers and the inclusion of 889 patients. Regarding the study’s limitations, it should be noted that it is a retrospective study, without the possibility of knowing the antiviral medication received by the patients during hospitalization. Furthermore, the pre-admission medication was not accessible for inclusion in the statistical analysis. Another limitation is the inability to monitor outcomes recorded during hospitalization. As a result, in the future, we recommend undertaking prospective studies in which the rate of thromboembolic events is assessed both at 30 days and three months following discharge.

## 5. Conclusions

Higher MLR, NLR, PLR, SII, SIRI, AISI, CONUT Score, and lower PNI values at admission highly predict DVT risk, APE risk, and mortality in COVID-19 patients, according to our data. Furthermore, PAD, malignancy, and tobacco, all predicted all outcomes, while CKD predicts APE risk and mortality but not DVT risk.

Given the high risk of thromboembolic events in COVID-19 patients and the inexpensive cost of these inflammatory and nutritional indicators, they can be used to classify admission risk groups, improve patient care, and establish predictive patterns. However, regarding the study’s limitations (being a retrospective study, without the possibility of knowing the antiviral medication received by the patients or the pre-admission medication), we recommend undertaking prospective studies in which the rate of thromboembolic events is assessed both at 30 days and three months following discharge.

## Figures and Tables

**Table 1:** Inflammatory and Nutritional Markers utilized in the study.

***Inflammatory Markers***				
Monocyte-Lymphocyte ratio (MLR)	$\frac{\mathrm{total}\;\mathrm{number}\;\mathrm{of}\;\mathrm{monocytes}}{\mathrm{total}\;\mathrm{number}\;\mathrm{of}\;\mathrm{lymphocytes}}$			
Neutrophil-Lymphocyte ratio (NLR)	$\frac{\mathrm{total}\;\mathrm{number}\;\mathrm{of}\;\mathrm{monocytes}}{\mathrm{total}\;\mathrm{number}\;\mathrm{of}\;\mathrm{lymphocytes}}$			
Platelets-Lymphocyte ratio (PLR)	$\frac{\mathrm{total}\;\mathrm{number}\;\mathrm{of}\;\mathrm{platelets}}{\mathrm{total}\;\mathrm{number}\;\mathrm{of}\;\mathrm{lymphocytes}}$			
Systemic Inflammatory Index (SII)	$\frac{\mathrm{total}\;\mathrm{number}\;\mathrm{of}\;\mathrm{neutrophils}\;\times \;\mathrm{total}\;\mathrm{number}\;\mathrm{of}\;\mathrm{platelets}}{\mathrm{total}\;\mathrm{number}\;\mathrm{of}\;\mathrm{lymphocytes}}$			
Systemic Inflammation Response Index (SIRI)	$\frac{\mathrm{total}\;\mathrm{number}\;\mathrm{of}\;\mathrm{neutrophils}\;\times \;\mathrm{total}\;\mathrm{number}\;\mathrm{of}\;\mathrm{monocytes}}{\mathrm{total}\;\mathrm{number}\;\mathrm{of}\;\mathrm{lymphocytes}}$			
Aggregate Index of Systemic Inflammation (AISI)	$\frac{\mathrm{total}\;\mathrm{number}\;\mathrm{of}\;\mathrm{neutrophils}\;\times \;\mathrm{total}\;\mathrm{number}\;\mathrm{of}\;\mathrm{platelets}\;\times \;\mathrm{total}\;\mathrm{number}\;\mathrm{of}\;\mathrm{monocytes}}{\mathrm{total}\;\mathrm{number}\;\mathrm{of}\;\mathrm{lymphocytes}}$			
***Nutritional Markers***				
Prognostic Nutritional Index (PNI)	[10 * serum albumin (g/dL)] + [0.005 × total number of lymphocytes/µL)]			
**CONUT Score**				
	Normal	Mild	Moderate	Severe
Albumin g/dL	≥3.50	3.00–3.49	2.50–2.99	<2.50
Score	0	2	4	6
Total Lymphocytes count /µL	≥1600	1200–1599	800–1199	<800
Score	0	1	2	3
Total Cholesterol mg/dL	≥180	140–179	100–139	<100
Score	0	1	2	3
Total Score	0–1	2–4	5–8	9–12

MLR = monocyte to lymphocyte ratio; NLR = neutrophil to lymphocyte ratio; PLR = platelets to lymphocyte ratio; SII = systemic inflammatory index; SIRI = systemic inflammation response index; AISI = aggregate index of systemic inflammation; PNI = prognostic nutritional index.

**Table 2:** The baseline characteristics data of all patients divided according to mortality.

Variables	All Patients n = 889	Survivors n = 746	Non-Survivors n = 143	*p* Value (OR; CI 95%)
Age mean ± SD (min-max)	70.5 ± 12.9 (21–101)	70.17 ± 12.74 (21–101)	72.18 ± 13.80 (24–97)	0.10
Male/Female sex no. (%)	474 (53.32%) 415 (46.68%)	397 (53.22%) 349 (46.78%)	77 (53.85%) 66 (46.15%)	0.89 (1.02; 0.71–1.46)
**Comorbidities and Risk Factors**				
AH, no. (%)	735 (82.67%)	615 (82.43%)	120 (83.91%)	0.66 (1.11; 0.68–1.80)
IHD, no. (%)	513 (57.70%)	427 (57.23%)	86 (60.13%)	0.52 (1.12; 0.78–1.62)
AF, no. (%)	252 (28.34%)	199 (26.67%)	53 (37.06%)	0.01 (1.61; 1.11–2.35)
CHF, no. (%)	215 (24.18%)	174 (23.32%)	41 (28.67%)	0.17 (1.32; 0.88–1.97)
MI, no. (%)	170 (19.12%)	133 (17.82%)	37 (25.87%)	0.02 (1.60; 1.05–2.44)
T2D, no. (%)	268 (30.14%)	223 (29.89%)	45 (31.46%)	0.70 (1.07; 0.73–1.58)
CKD, no. (%)	141 (15.86%)	108 (14.47%)	33 (23.07%)	0.01 (1.77; 1.14–2.74)
PAD, no. (%)	125 (14.06%)	91 (12.19%)	34 (23.77%)	0.0003 (2.24; 1.44–3.49)
Malignancy, no. (%)	74 (8.32%)	52 (6.97%)	22 (15.38%)	0.001 (2.42; 1.42–4.14)
Tobacco, no. (%)	256 (28.79%)	195 (26.13%)	61 (42.65%)	0.0001 (2.10; 1.45–3.04)
Obesity, no. (%)	146 (16.42%)	112 (15.01%)	34 (23.77%)	0.01 (1.76; 1.14–2.72)
Dyslipidemia, no. (%)	172 (19.34%)	132 (17.69%)	40 (27.97%)	0.004 (1.80; 1.19–2.7552)
**Nutritional Status**				
PNI, median [Q1–Q3]	42.1 [36.2–47.7]	43.83 [38.6–48.75]	32.05 [28.97–35.4]	<0.0001
CONUT Score, median [Q1–Q3]	3 [2,3,4,5]	3 [1,2,3,4]	7 [6,7,8,9]	<0.0001
Normal, no. (%)	192 (21.59%)	192 (21.59%)	-	0.001
Mild, no. (%)	397 (44.65%)	386 (51.74%)	11 (7.69%)	<0.0001
Moderate, no. (%)	259 (29.13%)	164 (21.98%)	95 (66.43%)	<0.0001
Severe, no. (%)	41 (4.61%)	4 (0.53%)	37 (25.87%)	<0.0001
**Laboratory Data**				
Hemoglobin g/dL median [Q1–Q3]	13.1 [11.2–14.4]	13.2 [11.4–14.49]	12.4 [10.2–14.15]	0.001
Hematocrit % median [Q1–Q3]	39.8 [34.3–43.8]	40.31 [34.95–43.95]	36.8 [30.92–42.4]	0.0002
Glucose mg/dL median [Q1–Q3]	114.9 [95–144]	113.8 [94.92–142]	124.4 [99–154.5]	0.006
Cholesterol mg/dL median [Q1–Q3]	177.3 [146–210.9]	180.1 [149.75–213.1]	158.5 [135.2–194.85]	<0.0001
Triglyceride mg/dL median [Q1–Q3]	119.1 [91.7–166.5]	120.25 [94–167.81]	117.3 [87.25–150.6]	0.06
Albumin, median [Q1–Q3]	3.48 [2.94–3.96]	3.64 [3.1–4]	2.8 [2.47–3.13]	<0.0001
GFR (mL/min/1.73 m^2^) median [Q1–Q3]	75.39 [56.44–92.24]	75.63 [56.08–91.84]	74.38 [58.7–93.35]	0.43
BUN mg/dL median [Q1–Q3]	70.2 [39.7–173.7]	63.84 [38.7–167.8]	134.2 [52.2–215.1]	<0.0001
Creatinine mg/dL median [Q1–Q3]	1.52 [0.89–5.91]	1.30 [0.88–5.78]	2.98 [1.1–6.9]	<0.0001
Neutrophils × 10³/µL median [Q1–Q3]	8.1 [5.53–12.43]	7.58 [5.27–11.28]	12.2 [8.79–16.27]	<0.0001
Lymphocytes × 10³/µL median [Q1–Q3]	1.32 [0.9–1.95]	1.5 [1.06–2.01]	0.66 [0.41–1.02]	<0.0001
Monocyte × 10³/µL median [Q1–Q3]	0.73 [0.5–1.09]	0.73 [0.5–1.06]	0.72 [0.45–1.14]	0.47
PLT × 10³/µL median [Q1–Q3]	237 [185.3–302]	235.7 [187–294]	245 [179.5–333.5]	0.18
Potassium median [Q1–Q3]	4.29 [3.85–5.16]	4.3 [3.86–5.05]	4.27 [3.79–5.59]	0.34
Sodium median [Q1–Q3]	139 [135–142]	139 [135.4–142]	138.1 [134.9–142]	0.11
MLR, median [Q1–Q3]	0.54 [0.32–0.91]	0.47 [0.31–0.79]	1.14 [0.66–1.68]	<0.0001
NLR, median [Q1–Q3]	6.63 [3.12–12.75]	5.38 [2.81–9.62]	19.74 [11.29–29.61]	<0.0001
PLR, median [Q1–Q3]	167.89 [114.9–280.2]	156.22 [110.37–238.16]	363.16 [244.87–616.93]	<0.0001
SII, median [Q1–Q3]	1462.5 [675.8–3205.02]	1235.42 [633.4–2240.9]	4623 [2319–8087.5]	<0.0001
SIRI, median [Q1–Q3]	4.65 [1.91–10.31]	3.58 [1.64–8.40]	12.07 [7.1–22.08]	<0.0001
AISI, median [Q1–Q3]	1072.12 [380.4–2603.4]	798.8 [354.1–2108.1]	3151.3 [1454.48–5823.5]	<0.0001
**Outcomes**				
DVT, no. (%)	191 (21.48%)	91 (12.19%)	100 (69.93%)	<0.0001 (16.73; 11–25.45)
APE, no. (%)	62 (6.97%)	24 (3.21%)	38 (26.57%)	<0.0001 (10.88; 6.27–18.88)
DVT APE, no. (%)	38 (4.27%)	11 (1.47%)	27 (18.88%)	<0.0001 (15.55; 7.51–32.2)
Length of hospital stay, median [Q1–Q3]	8 [6–11]	8 [6–12]	8 [6–12]	0.26

AH = arterial hypertension; IHD = ischemic heart disease; AF = atrial fibrillation; CHF = chronic heart failure; MI = myocardial infarction; T2D = type 2 diabetes; PAD = peripheral arterial disease; CKD = chronic kidney disease; MLR = monocyte to lymphocyte ratio; NLR = neutrophil to lymphocyte ratio; PLR = platelets to lymphocyte ratio; SII = systemic inflammatory index; SIRI = systemic inflammation response index; AISI = aggregate index of systemic inflammation; PNI = prognostic nutritional index; CONUT = controlling nutritional status; GFR = glomerular filtration rate; DVT = deep vein thrombosis; APE = acute pulmonary embolism.

**Table 3:** The AUC of the ROC curve, 95% confidence interval, sensitivity, and specificity of the inflammatory and nutritional markers.

Variables	Cut-Off	AUC	Std. Error	95% CI	Sensitivity	Specificity	*p* Value
**Deep Vein Thrombosis**							
**MLR**	0.78	0.824	0.020	0.785–0.863	77%	76.2%	<0.0001
**NLR**	9.63	0.836	0.017	0.802–0.870	77%	77.8%	<0.0001
**PLR**	230.67	0.802	0.018	0.766–0.839	72.8%	76.8%	<0.0001
**SII**	1890.45	0.805	0.019	0.768–0.842	79.1%	71.2%	<0.0001
**SIRI**	6.91	0.811	0.019	0.775–0.848	78%	72.3%	<0.0001
**AISI**	1605.4	0.784	0.020	0.745–0.823	71.2%	71.6%	<0.0001
**PNI**	40.12	0.803	0.017	0.769–0.837	66.8%	72.8%	<0.0001
**CONUT Score**	5.5	0.826	0.016	0.794–0.858	61.3%	85.8%	<0.0001
**Acute Pulmonary Embolism**							
**MLR**	0.81	0.766	0.034	0.699–0.832	71%	72.1%	<0.0001
**NLR**	13.67	0.801	0.031	0.740–0.861	67.7%	81%	<0.0001
**PLR**	207.06	0.734	0.036	0.664–0.804	74.2%	61.3%	<0.0001
**SII**	1839.91	0.761	0.034	0.694–0.828	75.8%	61.9%	<0.0001
**SIRI**	8.2	0.783	0.031	0.723–0.843	71%	70%	<0.0001
**AISI**	2769.85	0.750	0.034	0.684–0.816	61.3%	79.1%	<0.0001
**PNI**	35.22	0.864	0.025	0.814–0.914	82.6%	72.6%	<0.0001
**CONUT Score**	6.5	0.892	0.022	0.850–0.935	79%	89.5%	<0.0001
**Mortality**							
**MLR**	0.78	0.794	0.021	0.753–0.835	71.3%	74%	<0.0001
**NLR**	9.4	0.868	0.015	0.838–0.898	81.8%	74.4%	<0.0001
**PLR**	266.9	0.819	0.021	0.778–0.860	72%	81.1%	<0.0001
**SII**	2208.95	0.836	0.018	0.800–0.871	79.7%	74.4%	<0.0001
**SIRI**	7.47	0.801	0.019	0.764–0.839	72%	72.1%	<0.0001
**AISI**	1696.18	0.780	0.021	0.740–0.821	72%	70.9%	<0.0001
**PNI**	36.57	0.903	0.014	0.874–0.931	83.4%	84.6%	<0.0001
**CONUT Score**	4.5	0.913	0.012	0.889–0.937	92.3%	77.5%	<0.0001

MLR = monocyte to lymphocyte ratio; NLR = neutrophil to lymphocyte ratio; PLR = platelets to lymphocyte ratio; SII = systemic inflammatory index; SIRI = systemic inflammation response index; AISI = aggregate index of systemic inflammation; PNI = prognostic nutritional index; CONUT = controlling nutritional status.

**Table 4:** Univariate analysis of inflammatory and nutritional markers and outcomes.

	DVT	APE	Mortality
**Low-MLR vs.** **High-MLR**	47/595 (7.9%) vs. 144/294 (48.98%) *p* < 0.0001	18/613 (2.94%) vs. 44/276 (15.94%) *p* < 0.0001	42/595 (7.06%) vs. 101/294 (34.35%) *p* < 0.0001
**Low-NLR vs.** **High-NLR**	44/587 (7.5%) vs. 147/302 (48.68%) *p* < 0.0001	20/690 (2.9%) vs. 42/199 (21.11%) *p* < 0.0001	26/581 (4.48%) vs. 117/308 (37.99%) *p* < 0.0001
**Low-PLR vs.** **High-PLR**	52/581 (8.95%) vs. 139/308 (45.13%) *p* < 0.0001	16/523 (3.06%) vs. 46/366 (12.57%) *p* < 0.0001	40/645 (6.20%) vs. 103/244 (42.21%) *p* < 0.0001
**Low-SII vs.** **High-SII**	40/537 (7.45%) vs. 151/352 (42.9%) *p* < 0.0001	15/527 (2.85%) vs. 47/362 (12.98%) *p* < 0.0001	29/584 (4.97%) vs. 114/305 (37.38%) *p* < 0.0001
**Low-SIRI vs.** **High-SIRI**	42/547 (7.68%) vs. 149/342 (43.57%) *p* < 0.0001	18/597 (3.02%) vs. 44/292 (15.07%) *p* < 0.0001	40/578 (6.92%) vs. 103/311 (33.12%) *p* < 0.0001
**Low-AISI vs.** **High-AISI**	55/555 (9.91%) vs. 136/334 (31.44%) *p* < 0.0001	24/678 (3.54%) vs. 38/211 (18.01%) *p* < 0.0001	40/569 (7.03%) vs. 103/320 (32.19%) *p* < 0.0001
**Low-PNI vs.** **High-PNI**	52/519 (10.02%) vs. 139/370 (37.57%) *p* < 0.0001	17/700 (2.43%) vs. 45/189 (23.81%) *p* < 0.0001	22/644 (3.42%) vs. 121/245 (43.39%) *p* < 0.0001
**Low-CONUT Score vs.** **High-CONUT Score**	74/673 (11%) vs. 117/216 (54.17%) *p* < 0.0001	13/753 (1.73%) vs. 49/136 (36.03%) *p* < 0.0001	11/589 (1.87%) vs. 132/300 (44%) *p* < 0.0001

MLR = monocyte to lymphocyte ratio; NLR = neutrophil to lymphocyte ratio; PLR = platelets to lymphocyte ratio; SII = systemic inflammatory index; SIRI = systemic inflammation response index; AISI = aggregate index of systemic inflammation; PNI = prognostic nutritional index; CONUT = controlling nutritional status.

**Table 5:** Multivariate analysis for predictors of DVT and APE risk, and mortality during the hospitalization stay.

	Deep Vein Thrombosis			Acute Pulmonary Embolism			Mortality		
	OR	95% CI	*p*-Value	OR	95% CI	*p*-value	OR	95% CI	*p*-Value
**Age > 70** **CHF** **AF**	1.08	0.78–1.50	0.62	1.26	0.74–2.16	0.38	1.45	0.98–2.10	0.052
	1.14	0.85–1.51	0.36	0.88	0.50–1.56	0.67	1.14	0.84–1.55	0.38
	1.11	0.84–1.46	0.44	1.05	0.67–1.64	0.82	1.23	0.92–1.46	0.15
**MI**	0.96	0.65–1.43	0.87	0.78	0.39–1.52	0.47	1.35	0.89–2.04	0.14
**CKD**	1.19	0.78–1.82	0.40	1.95	1.07–3.55	0.02	1.77	1.14–2.75	0.01
**PAD**	2.10	1.52–2.91	<0.001	2.12	1.07–3.56	0.005	1.92	1.34–2.76	<0.001
**Malignancy**	2.70	1.65–4.42	<0.001	4.55	2.53–8.18	<0.001	2.42	1.42–4.14	0.001
**Tobacco**	2.01	1.44–2.81	<0.001	2.32	1.37–3.91	0.02	2.10	1.45–3.04	<0.001
**Obesity**	0.66	0.44–1.05	0.053	0.78	0.41–1.51	0.47	0.78	0.50–1.22	0.28
**Dyslipidemia**	1.06	0.70–1.59	0.38	1.42	0.77–2.62	0.25	1.19	0.76–1.85	0.44
**high-MLR**	11.19	7.68–16.29	<0.001	8.96	5.11–15.69	<0.001	6.89	4.64–10.23	<0.001
**high-NLR**	11.70	7.99–17.13	<0.001	10.50	5.86–18.8	<0.001	13.07	8.29–20.62	<0.001
**high-PLR**	8.36	5.82–12.02	<0.001	6.26	3.54–11.07	<0.001	11.04	7.34–16.62	<0.001
**high-SII**	9.33	6.35–13.71	<0.001	5.09	2.80–9.26	<0.001	11.42	7.36–17.72	<0.001
**high-SIRI**	9.28	6.34–13.58	<0.001	5.70	3.23–10.07	<0.001	6.66	4.47–9.92	<0.001
**high-AISI**	6.24	4.38–8.89	<0.001	5.98	3.49–10.24	<0.001	6.27	4.21–9.34	<0.001
**low-PNI**	5.40	3.78–7.71	<0.001	12.55	6.98–22.56	<0.001	27.58	16.84–45.19	<0.001
**high-CONUT Score**	9.56	6.67–13.71	<0.001	32.06	16.72–61.44	<0.001	41.28	21.8–78.19	<0.001

AF = atrial fibrillation; CHF = chronic heart failure; MI = myocardial infarction; PAD = peripheral arterial disease; CKD = chronic kidney disease; MLR = monocyte to lymphocyte ratio; NLR = neutrophil to lymphocyte ratio; PLR = platelets to lymphocyte ratio; SII = systemic inflammatory index; SIRI = systemic inflammation response index; AISI = aggregate index of systemic inflammation; PNI = prognostic nutritional index; CONUT = controlling nutritional status.

## Data Availability

Not applicable.
